# Community-based malaria surveillance in Cambodia using a mobile reporting app for village malaria workers: An implementation case study

**DOI:** 10.1371/journal.pdig.0001626

**Published:** 2026-09-03

**Authors:** Pengby Ngor, Huy Rekol, Siv Sovannaroth, Po Ly, Eng Thyda, Sok Kimleng, Yoem Rattana, Ou Vunsokserey, Hem Vanna, Ran Sany, Chang Pumeoung, Steven Mellor, Rady Try, Pascal Ringwald, Richard J. Maude, Lisa J. White

**Affiliations:** 1 National Center for Parasitology, Entomology and Malaria Control, Phnom Penh, Cambodia; 2 Mahidol Oxford Tropical Medicine Research Unit, Faculty of Tropical Medicine, Mahidol University, Bangkok, Thailand; 3 The Open University, Milton Keynes, United Kingdom; 4 Independent Consultant, Phnom Penh, Cambodia; 5 WHO Mekong Malaria Elimination Programme, Phnom Penh, Cambodia; 6 Nuffield Department of Medicine, Centre for Tropical Medicine and Global Health, University of Oxford, Oxford, United Kingdom; 7 Model Health Ltd, Brixham, United Kingdom; The University of Sheffield, UNITED KINGDOM OF GREAT BRITAIN AND NORTHERN IRELAND

## Abstract

To support malaria elimination, Cambodia implemented a locally developed, nationally integrated, community-based mobile digital surveillance system enabling real-time, case-based detection, reporting and response. This implementation case study describes the development, national deployment and operational performance of an Android-based mobile app used by Village Malaria Workers (VMWs) and Mobile Malaria Workers (MMWs) integrated within the national Malaria Information System (MIS). Developed collaboratively with VMWs, the system enables real-time, geolocated case reporting with offline functionality, built-in validation checks, centralized device management and automated analytics. Evaluation employed three data sources: routine surveillance data (2018–2024), a contextual survey of VMW/MMWs in three high-risk provinces (2024), and a national technical usability survey (2025). Outcomes included reporting timeliness, completeness, surveillance performance indicators aligned with the national malaria elimination framework and user experience. Following national implementation in 2017− 2018, data completeness and timeliness improved. Between 2018 and 2024 reporting coverage increased from 19% to 99% and data completeness has remained ≥99% since 2019. Case notification within 24 hours increased from 2% to 99% and foci investigation within 7 days from 0% to 98%. Malaria testing rates expanded from 135,664 to 715,277 per year. Intensified surveillance was concurrent with a decline in active malaria foci from 129 in 2020 to 0 in 2024. A contextual evaluation including 88 VMW/MMWs showed strong support for surveillance activities, including reporting and data analysis at the community level. The technical evaluation survey found that 94% (904/966) of VMWs were very satisfied/satisfied with the app, with technical problems experienced never/rarely by 82% (795/966). Key operational challenges included intermittent internet connectivity, limited electricity supply, and transport constraints, although offline functionality and data synchronization prevented data loss. Cambodia’s experience demonstrates the feasibility and sustainability of large-scale digital surveillance in resource-constrained settings and highlights the importance of integration, training and sustainability through local ownership.

## Author summary

Comprehensive and timely malaria surveillance is an essential component for achieving malaria elimination in Cambodia. The implementation of the VMW app as part of the wider MIS substantially strengthened malaria surveillance in Cambodia. Previously, geographical remoteness, limited transport and communication barriers caused delays in case notification and slowed public health responses. The VMW mobile app has addressed these challenges by enabling individual case-based, real-time, geolocated reporting and improving coordination between VMWs and health authorities. Locally developed and maintained using open-source software, the system is sustainable and adaptable to elimination and post-elimination settings. The app is also amenable to expansion or repurposing to other disease surveillance needs. This experience illustrates that digital health solutions to support community-based surveillance can be sustainably deployed in low-resource settings to positively impact public health.

## Introduction

Around half of the world’s population are at risk of malaria, a parasitic disease caused by *Plasmodium spp.* and transmitted by *Anopheles* mosquitoes [1]. Historically, border regions of the Greater Mekong Subregion (GMS) have been a focus for the emergence of drug-resistant *Plasmodium falciparum* [2]. Resistance to chloroquine, sulfadoxine, pyrimethamine, quinine and mefloquine originated in the GMS, spreading globally and undermining treatment efficacy.

In 2006 the World Health Organization recommended artemisinin-based combination therapies (ACTs) as first-line malaria treatment, contributing to substantial reductions in burden and mortality over the next decade [1]. However, in 2008 delayed parasite clearance following artemisinin was detected in western Cambodia [3]. The subsequent emergence of resistance to multiple ACT partner drugs led to high clinical failure rates, raising concerns that malaria would become untreatable [4–6]. In response, the World Health Organization (WHO) developed the Global Plan for Artemisinin Resistance Containment [7], aiming to restrict the problem to the Cambodia–Thailand border area. However, it became clear that the issue was regional across the GMS. Moreover, the potential for wider dissemination of multidrug-resistant parasites threatened a global health crisis. This triggered the WHO Emergency Response Framework [8], and ultimately a commitment from GMS countries to target malaria elimination by 2030 [9,10]. Progress towards this ambitious goal has been substantial; China has been certified malaria-free [1], while Cambodia, Thailand, the Lao People’s Democratic Republic (PDR) and Viet Nam have achieved marked reductions in malaria cases and mortality [1,10–14].

In Cambodia, intensified efforts to contain drug resistance and achieve malaria elimination reduced cases from 106,228 in 2010 to 355 in 2024, with no malaria deaths reported since 2018 [1,13]. Elimination activities are led by the National Center for Parasitology, Entomology and Malaria Control (CNM) under the National Strategic Plan for Malaria Elimination (2011–2025) and elimination action frameworks [15,16]. Key strategies include the ‘1-3-7’ approach, requiring case notification within one day, investigation within three days and foci response within seven days [17–20], alongside integrated drug efficacy surveillance (iDES) [21] and risk stratification to guide targeted interventions [22–25]. However, these strategies cannot be implemented without real-time, case-based surveillance at the point of care [26–28]. Hence, elimination-competent surveillance is a core intervention [9,26].

Malaria in Cambodia is concentrated in rural, remote, and border regions, often among mobile and migrant populations, and community-based approaches have been central to elimination efforts [29]. The village malaria worker (VMW) network, established in 2004, provides diagnosis, treatment, prevention and health education in underserved areas, while mobile malaria workers (MMWs) serve mobile and migrant populations. The aim is to deliver malaria services to the entire population of endemic regions [16]. In 2015, a web-based Malaria Information System (MIS) was launched, but VMWs continued to submit paper records, which were manually compiled into monthly reports at health centers [30,31]. This process failed to deliver the data completeness and timeliness needed to support elimination activities or coordinate outbreak responses [13]. Moreover, training and supervision for surveillance activities were impaired by the wide geographical spread of VMWs, their limited availability, and insufficient capacity to monitor interventions or provide VMWs with feedback on malaria trends in their areas.

**Fig 1:**
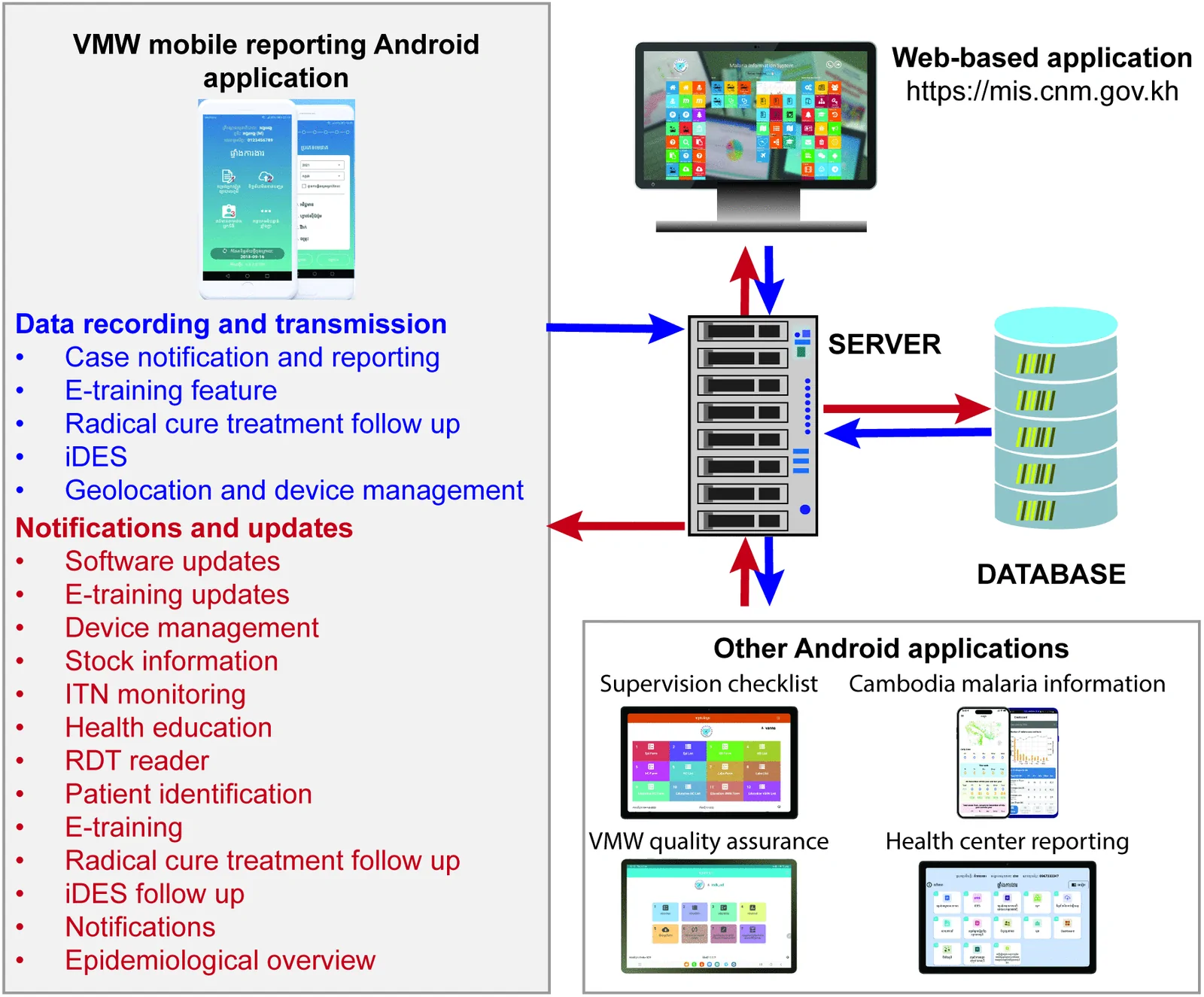
Village Malaria Worker mobile app functionality and integration into the Malaria Information System. iDES, integrated drug efficacy surveillance; ITN, insecticide treated bed net; RDT, rapid diagnostic test. Source CNM/MIS.

Since most malaria cases were diagnosed and treated in the community, it was clear that the primary bottleneck in Cambodia’s surveillance system was paper-based reporting through the VMW network [29,30,32]. Mobile health (mHealth) strategies were emerging as promising solutions in low-resource settings [33–35]. Hence, in 2017, CNM introduced an upgraded MIS, featuring a web-based platform integrated with Android-based mobile applications enabling VMWs and health center staff to report geolocated malaria cases directly from the field, supporting real-time data submission and analysis (Fig 1) [36,37]. The system was designed with the following aims:

- Reduce reporting delays to provide real-time data and improve accuracy to enable early outbreak detection, rapid response and effective risk stratification.
- Support patient management through scheduling and reminders, aiding follow-up and detection of potential drug resistance.
- Strengthen VMW capacity via integrated e-learning modules for continuous training.
- Provide timely feedback on local malaria trends, empowering VMWs and health staff to take informed action in case management, prevention, and elimination.

Notably, the system is deployed nationally at scale to all VMWs and MMWs and health centers across malaria-endemic regions. The aim is to identify, treat, classify and follow-up every malaria case in Cambodia while detecting outbreaks, monitoring resistance, strategically deploying resources, evaluating the impact of interventions and updating skills and knowledge (Fig 1). The system also aligns with WHO requirements for malaria-free certification [38,39].

Despite growing interest in mHealth technologies in low- and middle-income countries, important evidence gaps remain [40–43]. Previous reviews have noted that much of the mHealth literature consists of small-scale pilot projects, process evaluations or studies focused primarily on user acceptability, with relatively few formal evaluations demonstrating impacts on health system performance or population-level outcomes [33,40,42–44]. Furthermore, many published interventions have not progressed beyond pilot implementation, leaving limited evidence regarding sustainability, integration within national health information systems and operation at scale [33,40,45,46]. Reporting of implementation challenges and contextual factors has also been inconsistent, particularly regarding connectivity and reflexivity, making it difficult to understand how digital tools perform under routine program conditions or their adaptation as operational needs evolve [43,47,48].

This observational, descriptive study helps address these gaps by describing the development, national deployment and operational performance of a fully integrated mobile surveillance system supporting Cambodia’s malaria elimination program. Unlike many mHealth interventions that remain isolated pilot projects, the VMW mobile application is embedded within the national MIS, supports routine real-time, case-based surveillance across the country and has been sustained through domestic technical capacity and ownership. Combining routine surveillance data with contextual and technical evaluations, this study aims to assess usability and acceptability, surveillance timeliness, reporting performance and operational implementation under real-world conditions. As countries move from malaria control towards elimination, such evidence is needed to inform the design, scale-up and long-term sustainability of digital surveillance systems.

## Methods

### Ethics statement

The work was undertaken as part of routine surveillance strengthening activities led by CNM, including routinely collected programmatic data and surveys with VMWs regarding their professional use of a government-deployed malaria reporting application. All participants were VMWs responding in their official capacity, and participation in surveys was voluntary and anonymized. Patient-level identifiers or personal health information were not available or analyzed. In accordance with national regulations, formal ethical approval and written informed consent were not required. The study adhered to applicable institutional, national and international data protection standards.

### Study setting and intervention

Malaria elimination activities in Cambodia are coordinated by CNM through the national MIS, a web-based surveillance platform integrating malaria case management, epidemiological surveillance, entomological surveillance, iDES, supply chain management and device management (Fig 1). The MIS is integrated into the national health management information system and supports real-time reporting, analysis and visualization of case-based surveillance data.

To strengthen community-based surveillance, CNM developed and implemented an Android-based mobile app for VMWs and MMWs. The app enables individual case-based, geolocated reporting directly from villages and remote transmission areas. Data entered in the VMW app can be stored locally and synchronized with the central MIS when internet connectivity is available. The VMW app is integrated with the MIS web-based portal, built on the Laravel PHP framework with a Microsoft SQL database backend (Fig 1). Access is role-based and restricted according to user responsibilities. VMWs can only enter and review individual patient data relevant to their assigned communities and analytic outputs of the MIS contain anonymized or aggregated patient data. Supervisory staff have access appropriate to their administrative level. System administration and access approval are managed by authorized CNM personnel.

### Development of the VMW app

The VMW mobile app was developed in-house by the CNM MIS technical team in collaboration with implementation partners with technical support from Mahidol Oxford Tropical Medicine Research Unit (MORU). A human-centered design approach guided the process, prioritizing VMW needs, preferences and workflows to develop an intuitive and efficient tool [48,49].

In March/April 2017, consultations with CNM, provincial health departments and other key stakeholders were conducted to identify user needs, field constraints and practical design considerations. Emphasis was placed on ensuring the app would be intuitive and functional for VMWs operating in remote settings. Simultaneously, a detailed review of existing data flows within the malaria surveillance and reporting systems was conducted to identify redundancies, gaps and opportunities for integration.

System design and architecture development was conducted from May to July 2017. The VMW mobile app was developed using Android Studio and programmed in Java to ensure compatibility with the Android tablets and smartphones commonly used by VMWs. The app was built to be fully compatible with the existing MIS and included real-time data synchronization and dashboard-based visualization features to improve data access and utility. A modular system architecture was created, allowing future integration of additional components, such as iDES, insecticide-treated bed net distribution and rapid diagnostic test reporting, in line with the evolving national malaria strategy. Data standards and reporting formats were developed to ensure consistency with national and WHO guidelines [38,50]. A key technical feature was the integration of mobile device management to support remote device tracking, software version control and automatic updates.

Structured testing within CNM assessed performance and field readiness using scenario-based test cases that evaluated key functions, such as data entry, offline capture, MIS synchronization and automated alerts for common and outlier conditions. This allowed technical issues to be identified, documented and resolved prior to pilot testing with 27 active VMWs in Kampong Cham and Kratie provinces [51]. The app was used for routine malaria surveillance, assessing usability, reliability and contextual appropriateness, with observational data and user feedback highlighting barriers such as connectivity limitations or device compatibility issues. These insights informed further refinement prior to national rollout [51]. The combined approach of controlled internal testing and field-based evaluation provided a comprehensive verification of app functionality and usability.

Following deployment, continued development was informed by real-time performance monitoring, identification of user challenges and documentation of lessons learned. A dedicated helpdesk was established to provide technical support, while system documentation and a routine maintenance schedule were put in place to support sustainability. Continuous improvements and feature enhancements are guided by the evolving needs of users and the malaria control program. This iterative, participatory development process aimed to deliver a mobile health app that is responsive to both the technical and operational realities of community-based malaria elimination efforts.

### Application features

The VMW mobile app enables real-time data entry at the point of care, allowing immediate logging of malaria cases and eliminating the need for manual transcription. Through secure authentication, authorized users can access reporting functions and surveillance analytics. Data are stored on the device and transmitted securely to a central server once an internet connection is available, ensuring confidentiality and data integrity (Fig 1).

The user interface uses icon-based navigation to accommodate varying literacy and digital literacy levels (see S1 File for screenshots). Standardized data entry, through predefined options and drop-down menus, ensures consistency and reduces errors. Invalid or inconsistent inputs trigger alerts, prompting correction before submission. CNM staff conduct regular data quality checks to identify errors and provide targeted support to VMWs.

The app provides VMWs with real-time analytics on local malaria trends, reinforcing the value of their data. It also sends automated reminders, including follow-up prompts for patient care. For *Plasmodium vivax* cases requiring referral for radical cure, the app alerts VMWs if a referral is incomplete. Unique patient identifiers enable cases to be followed-up and the identification of recurrences, supporting early detection of drug resistance or suboptimal case management. Integrated support features include e-learning modules for continuous training, remote and in-person technical support, cost-free text messaging for communication with health centers and multi-language functionality to enhance accessibility.

### VMW Training and supervision

During nationwide deployment between November 2017 and March 2018, a total of 6,400 VMWs were trained in the use of the mobile app and associated system functions. The program comprised four key components: initial training, refresher training, on-the-job support and continuous learning through an e-training platform.

Initial training sessions were standardized to two days, with up to 250 participants per session, with two VMWs from each village. Sessions introduced app features, data entry protocols and reporting workflows, using interactive modules led by CNM and partner trainers. VMW performance was assessed through scenario-based exercises.

Annual refresher training addresses knowledge gaps, reinforces learning and introduces software updates. CNM trains provincial- and operational district-level staff alongside health center personnel who are in turn responsible for training local VMWs, promoting consistency and decentralized capacity building. Augmenting this approach, on-the-job training provides targeted support for VMWs with limited digital experience. Supervisors accompany VMWs during field visits, enabling real-time guidance and practical application of skills in authentic settings. Every VMW has access to ongoing support on data reporting through monthly meetings or messaging the health center. Any queries that cannot be addressed at that level are escalated to the operational district malaria supervisor, provincial malaria supervisor or civil service organization responsible for the health center and then, if necessary, to CNM.

Recognizing VMWs’ limited time availability, self-paced e-learning modules are embedded within the app, including videos, slides and quizzes which are updated as issues emerge or to reflect policy changes. Thus, VMWs have continuous access to training and a shared responsibility for maintaining their knowledge and skills. This ensures all VMWs are up-to-date and consistently trained while reducing the time needed away from their other responsibilities. Beyond the initial deployment of the VMW app, onboarding of new VMWs follows a similar pattern with in-person training, supported by field visits and e-learning. This structured, multi-modal approach promotes long-term engagement with digital surveillance.

### Device and software deployment

**Fig 2:**
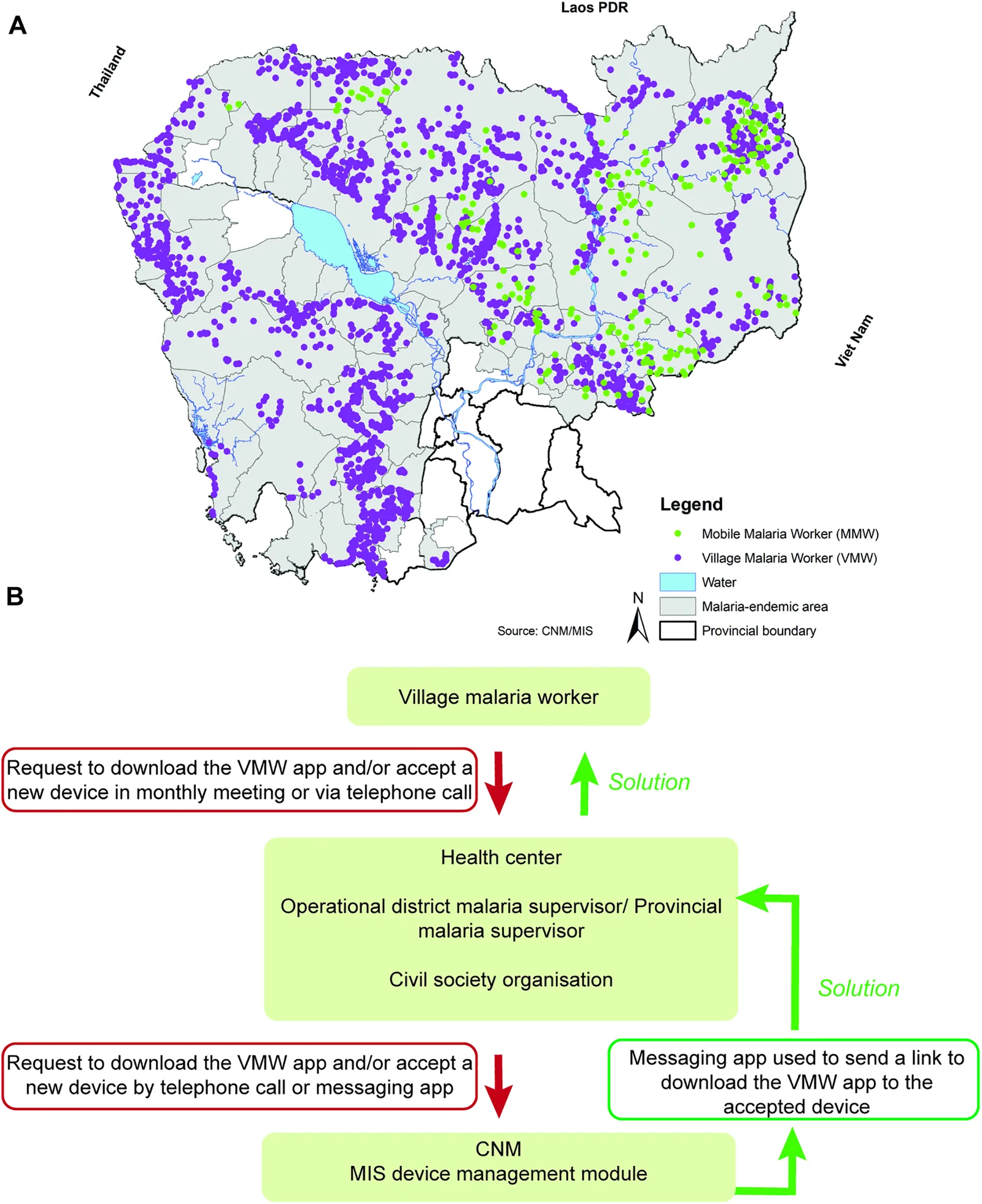
Deployment of the Village Malaria Worker mobile app. (A) distribution of trained VMWs and MMWs in the use of the mobile app; (B) support for installation of the VMW app. The basemap shapefile was obtained from the GADM database of Global Administrative Areas, version 4.1 (https://gadm.org) and the map design used Esri (World Countries/Administrative Boundaries) in ArcGIS Pro. VMW and MMW location data are from CNM/MIS.

Between 2017 and 2021, CNM distributed Android smartphones pre-installed with the VMW mobile app, supported by Global Fund financing. Devices were configured to meet technical requirements and ensure reliable field performance. However, VMWs were permitted to use their own technically suitable smartphones on request. From 2023, VMWs were encouraged to use personal smartphones, reflecting increased mobile ownership and aiming to reduce reliance on external funding, thereby enhancing long-term sustainability but increasing the range of devices in use. Older spare devices are often retained in case of damage or loss and some VMWs now own tablets as well as phones and prefer the flexibility to use both. As of 5^th^ June, 2025, there were 10,015 active mobile devices of 519 different models distributed across 6,840 VMWs and MMWs in 3,603 villages (Fig 2A).

To prevent disruptions from device loss, damage or malfunction, a device management module was integrated into the MIS. This system tracks device location and performance, streamlining administration and maintenance. It also permits remote device verification and access management. Fig 2B indicates the process for first installation on a new device. While comparable to commercial mobile device management platforms, this in-house solution was designed to be cost-effective across Cambodia’s large VMW network. Data security and access control are reinforced through an integrated user authentication system. Each VMW receives a unique ID linked to their village, enabling access upon administrator approval. After initial login, reauthentication is not required. Devices are also configured for automatic software updates, ensuring continued access to the latest app features without user intervention.

### Evaluation framework and data sources

This implementation case study drew upon three distinct data sources: (1) routine surveillance data collected from the VMW app and reported through the national MIS, (2) contextual evaluation surveys assessing the operational environment and surveillance capacity of VMWs and MMWs, and (3) a technical evaluation survey assessing app usability, performance and user satisfaction. Each data source served a different purpose and was analyzed separately. No formal hypothesis testing was undertaken because the objective of the study was to describe the development, implementation, performance and operational experience of a national community-based digital malaria surveillance system.

(1) **Routine surveillance**

Routine surveillance data were used to assess surveillance performance and programmatic outcomes. De-identified aggregated data were extracted from the national MIS, which receives case-based surveillance information submitted through the VMW mobile app and associated reporting systems (Fig 1). Surveillance indicators were examined from January 2018 to July 2025, corresponding to the period following national implementation.

Outcomes reflected 1-3-7 framework targets which require data timeliness and completeness. VMW engagement and productivity were assessed through the number of diagnostic tests conducted. Programmatic impact on malaria burden was indicated by clearance of malaria foci. These outcomes aligned with the National Malaria Elimination Action Framework, WHO malaria surveillance recommendations and the requirements for achieving malaria elimination certification as per WHO technical guidelines [16,26,38,50].

Data quality assurance processes include built-in features in the VMW app and MIS, such as mandatory fields and restricted data entry options, automated validation checks, and real-time alerts for anomalous values. Human-led validation includes supervisor review processes, routine monitoring of reporting completeness and timeliness versus paper-based auditing, annual refresher training and ongoing supportive supervision, field-based troubleshooting and corrective training when data quality issues are detected. These data completeness and validation measures are key for demonstrating malaria elimination in accordance with the WHO certification process [38,39]. Routine surveillance data were analyzed descriptively. Annual frequencies, proportions and temporal trends were calculated to assess surveillance timeliness, completeness and programmatic performance over time.

(2) **Contextual evaluation survey**

A contextual evaluation survey was conducted to assess factors influencing surveillance performance, including infrastructure availability, connectivity, reporting practices, training, supervision, commodity availability, surveillance knowledge and confidence in data use.

CNM conducts biannual contextual evaluation surveys as part of routine programmatic monitoring. The most recent survey was conducted between 1 August and 1 December 2024 in Battambang, Kampong Speu and Mondul Kiri provinces. This was a purposive sample to evaluate VMW app operational effectiveness in areas with high-risk, forest-exposed populations. Battambang (along the Thai border) and Mondul Kiri (along the Vietnamese border) have historically been high-transmission, forested “hotspot” zones for *P. falciparum*. Additionally, Kampong Speu and Mondul Kiri were implementing localized malaria elimination intensification plans, which require highly responsive surveillance. Thus, the survey was designed to provide highly specific, quantitative and operational feedback in the most challenging settings within Cambodia.

All VMWs/MMWs were invited to participate and 88 consented. Participants completed a structured questionnaire examining access to mobile devices, internet connectivity, electricity supply, reporting practices, stock availability, knowledge of surveillance procedures, training received and confidence in surveillance-related activities. These domains reflect programmatic objectives specific to the intensification activities and were informed by spontaneous feedback received from VMWs through the helpdesk or CNM troubleshooting processes. Surveys were completed remotely through the VMW app and uploaded when internet connectivity was available. To avoid bias, supervisors did not have access to the survey responses whereas researchers had access to de-identified data amalgamated within the MIS; participants were therefore anonymized. Survey responses were analyzed descriptively with the aim of identifying operational barriers and opportunities to strengthen surveillance implementation and support systems.

(3) **Technical evaluation**

A national technical survey was conducted to assess usability, acceptability and operational performance of the VMW mobile app in August 2025. All operational VMWs were invited to complete the survey, with 996 consenting to participate. Surveys were completed remotely through the VMW app and uploaded when internet connectivity was available. To avoid bias, supervisors did not have access to the survey responses whereas researchers had access to de-identified data amalgamated within the MIS; participants were therefore anonymized. The survey assessed overall satisfaction, perceived app speed, frequency of technical problems, ease of navigation, training requirements and operational challenges associated with app use. These domains were informed by technical feedback received from users spontaneously through the helpdesk or CNM troubleshooting processes. Technical survey responses were summarized descriptively.

## Results

### Routine surveillance outcomes

**Fig 3:**
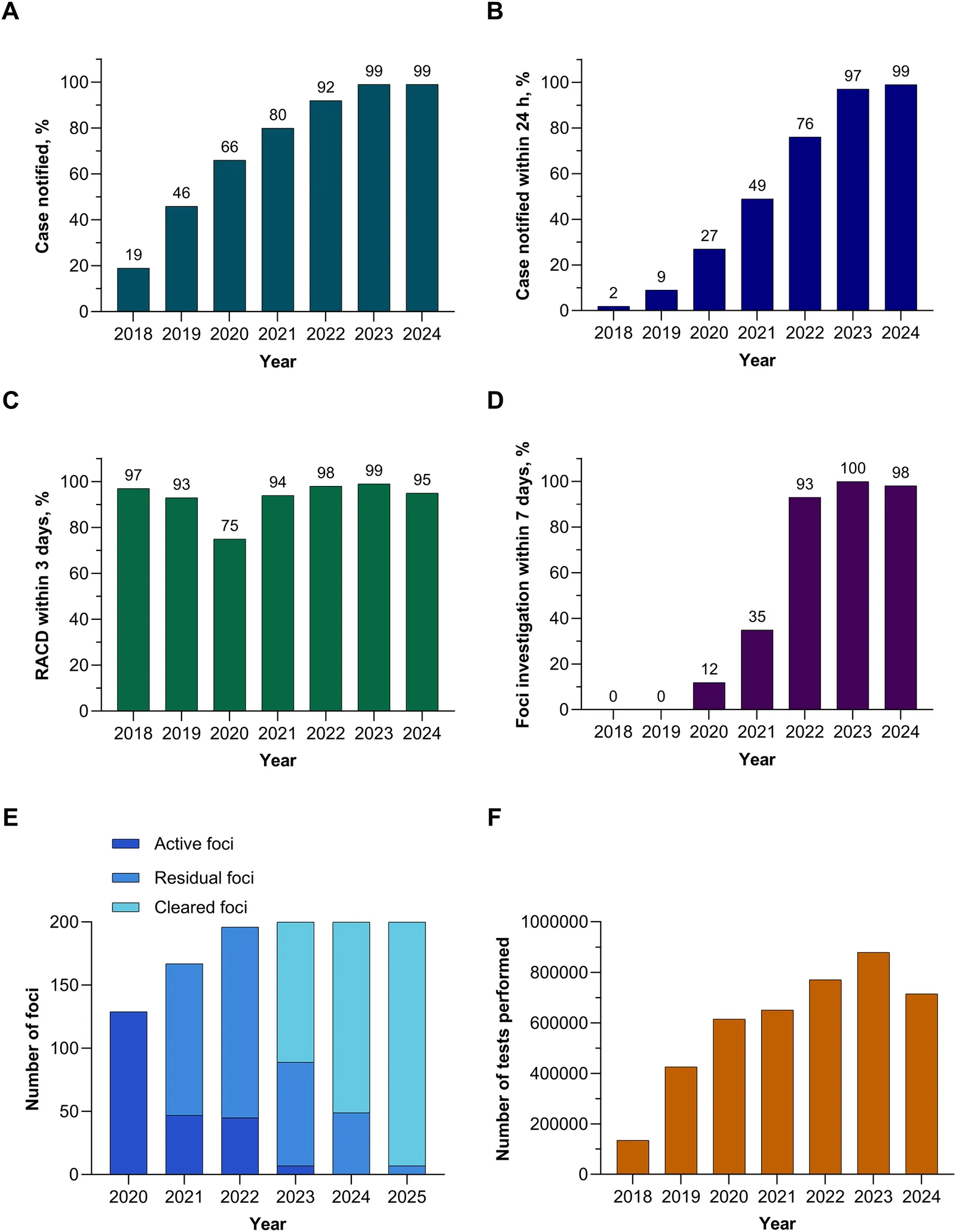
Surveillance outcomes supported by mobile case-based real-time reporting by Village Malaria Workers. **(A)** VMW percentage of cases notified; **(B)** VMW percentage of notified cases within 24 hours; (C) percentage of reactive case detection (RACD) completed within 3 days; (D) percentage of foci investigations completed within 7 days; (E) number of *P. falciparum* foci which were active, residual or cleared up to and including 7^th^ July, 2025 (foci-based activities commenced in 2020); (F) number of diagnostic tests performed by VMWs. Source CNM/MIS.

The primary advantage of mobile reporting is the early detection of malaria cases, enabling prompt treatment and reducing severe illness and mortality. From 2018 to 2024, reporting coverage increased from 19% to 99% of cases (Fig 3A), and the proportion of cases notified within 24 hours rose from 2% to 99% (Fig 3B). Case investigation improved over the same timeframe, with VMWs routinely capturing contextual data such as travel history and household exposure, essential for identifying infection sources and understanding transmission. Data completeness has remained ≥99% since 2019, compared to 75% with paper records in 2016. The availability of real-time data supported rapid response times. By 2024, reactive case detection (testing and treating household contacts and neighbors) was completed within 3 days in 95% of cases (Fig 3C), and foci investigations were completed within 7 days for 98% of cases (Fig 3D). These rapid responses are vital for interrupting transmission and containing outbreaks. More timely and effective interventions were associated with a decline in active malaria foci from 129 in 2020 to 0 in 2024 (Fig 3E). Over this period malaria diagnostic testing rates have expanded, increasing from 135,664 per year in 2018 to 715,277 in 2024, reflecting the sustained engagement and productivity of VMWs (Fig 3F).

### Contextual evaluation survey

**Fig 4:**
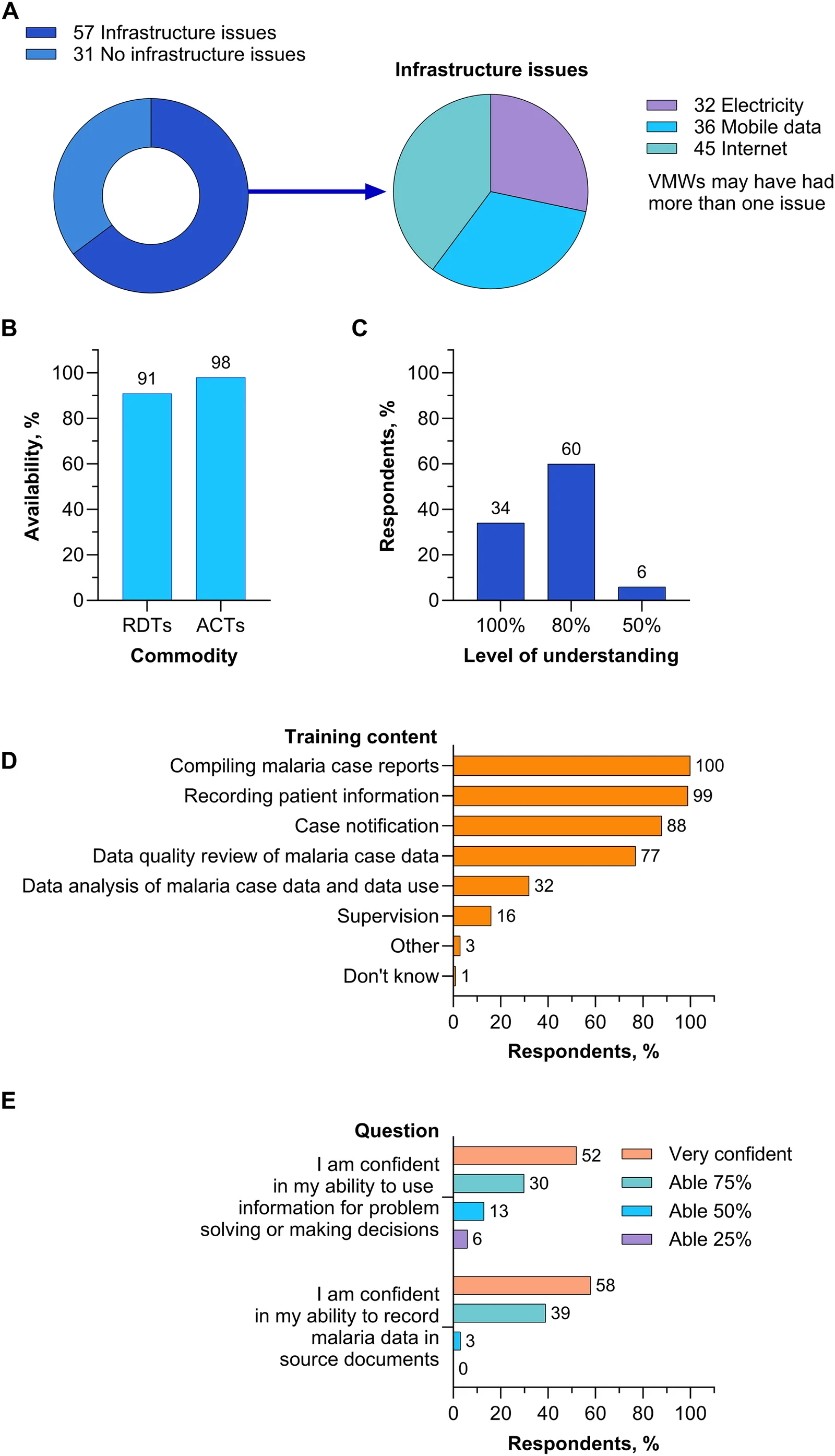
Contextual user evaluation of the Village Malaria Worker mobile app for malaria surveillance. **(A)** Infrastructure availability and issues; (B) proportion of VMWs with stock availability for rapid diagnostic tests (RDTs) and artemisinin-based combination therapies (ACTs) over the previous 6 months; (C) current understanding of data elements/ variables/ fields recorded for malaria surveillance; (D) self-reported content of training delivered over the past year; (E) self-reported confidence in recording malaria data and using information for problem solving and decision making. Source CNM/MIS.

In-depth biannual evaluation surveys are conducted to assess VMW app user contextual experience for malaria surveillance, targeted at operationally critical areas. The most recent survey (1 Aug–1 Dec 2024) included 88 VMWs/MMWs across Battambang, Kampong Speu and Mondul Kiri (Fig 4). The survey confirmed that all respondents knew that malaria case reporting was mandatory and that case-based data were required, with 98% (86/88) aware of national surveillance guidelines. All respondents knew they needed to have a mobile phone and a patient register book, with these consistently available over the last 30 days for 95% (84/88) of VMWs. Three mobile phones were non-functional within the last 30 days, underscoring the importance of the MIS device management module which alerts CNM staff to issues requiring device repair or replacement. All respondents confirmed they had access to troubleshooting support.

Connectivity remains a challenge, with 65% (57/88) of VMWs reporting at least one issue (internet, data or electricity) in the past 30 days (Fig 4A). The app mitigates this by storing data offline and synchronizing automatically once connected. Thus, though reporting can be delayed, data are preserved for later upload from the VMW app to the MIS. Also, as a standard suite of surveillance analytics is generated in the MIS overnight, download times and data transfer requirements to the VMW app are minimized. Most VMWs had uninterrupted access to diagnostics and treatments (Fig 4B), supported by MIS stock tracking and alerts based on VMWs commodity consumption data.

All except one VMW used the VMW app for data reporting. Standardized CNM paper records were also used by 98% (86/88) of VMWs; these are not uploaded but are required for auditing purposes to demonstrate data integrity for malaria-free certification as specified by WHO [38,50]. They also allow investigation of data anomalies by CNM.

Knowledge gaps were noted, with imperfect understanding of the data elements/fields recorded for malaria surveillance (Fig 4C). Also, 11% (10/88) of respondents believed job aids and standard operating procedures required improvement, 15% (13/88) had not received a job aid and 6% (5/88) thought recording data was unclear or complicated. However, only 2% (2/88) of VMWs were unsure of who to contact for troubleshooting and support. Knowledge of the reporting process was reasonable; 88% (77/88) of respondents knew case-based data were reported and 97% (85/88) of respondents confirmed that they reported malaria case information immediately. However, 19% (17/88) were unaware that zero case reports are also required.

Consistent with the annual training schedule, all respondents received surveillance training in the previous year (Fig 4D). All VMWs felt able to record malaria data and use information to problem solve, with around half feeling very confident in their abilities (Fig 4E). However, tailored on-the-job training for some individuals would improve their confidence.

### Technical evaluation

**Fig 5:**
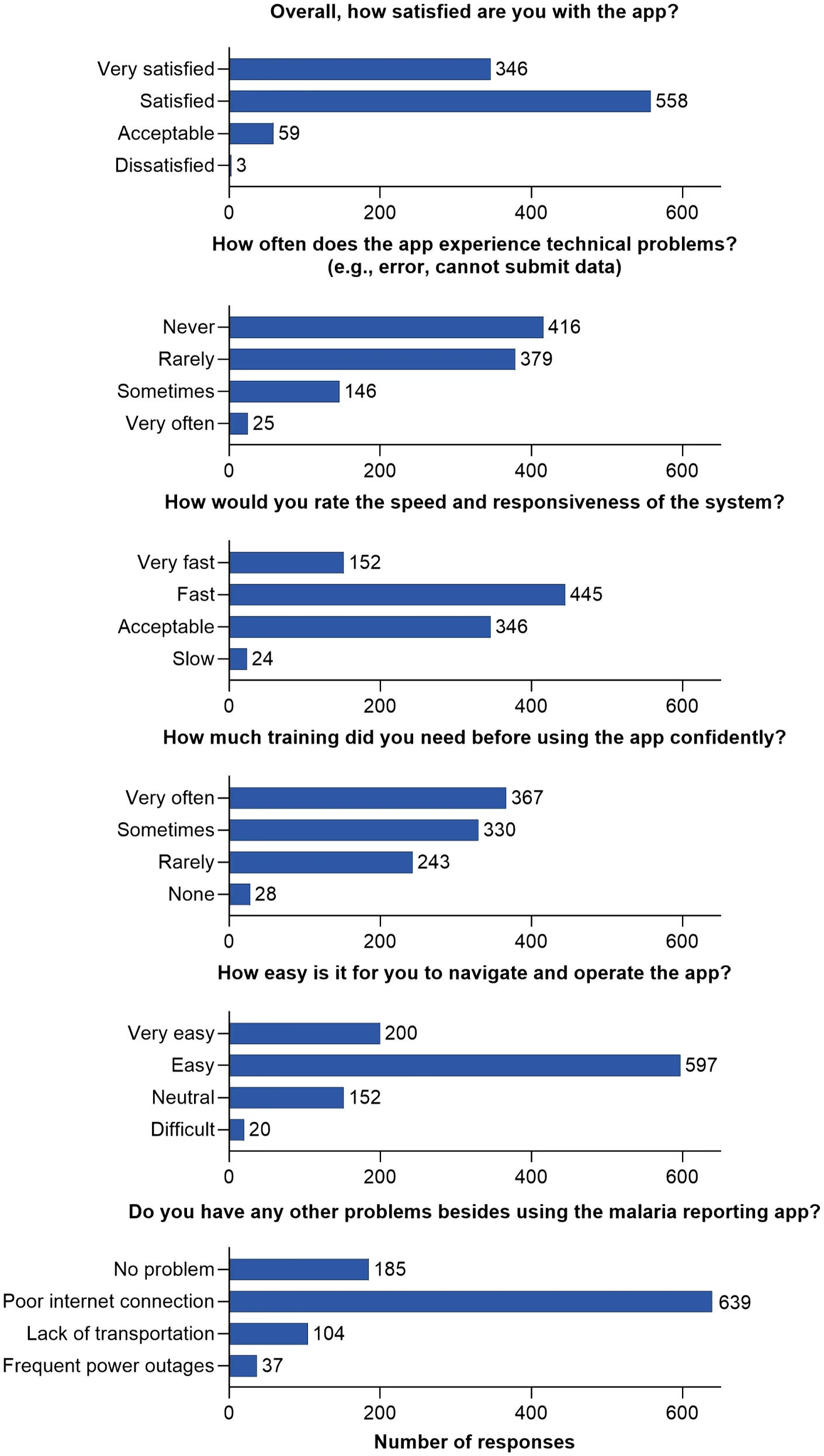
Technical evaluation of the Village Malaria Worker mobile app. Source CNM/MIS.

A technical evaluation survey including 996 VMWs conducted in August 2025 (Fig 5) found that 94% (904/966) were very satisfied or satisfied with the app and technical problems were experienced never or rarely by 82% (795/966). Most (62%; 597/967) VMWs found the app was very fast/fast, and 98% (943/967) thought that the app speed was at least acceptable. The importance of continued training was highlighted, with training required very often or sometimes by 72% (697/968) of VMWs before they became confident, though just 2% (20/969) expressed difficulty in navigating or operating the app. Associated challenges with the app were most commonly a poor internet connection (66%; 639/965), lack of transportation to access cases or follow-up (11%; 104/965) and intermittent electricity supply (4%; 37/965).

## Discussion

This study demonstrates that integrating mobile, real-time reporting into Cambodia’s community-based malaria surveillance system enhanced the timeliness, completeness and operational utility of case-based data. The near-universal reporting coverage (99%), rapid notification within 24 hours, and high compliance with 1-3-7 response timelines indicate that the system is functioning as intended and is capable of supporting malaria elimination. These improvements are not merely technical gains; they permit faster case management, more efficient reactive case detection and timely foci investigation, which together are critical for interrupting transmission. Improvements in surveillance performance should be interpreted within the broader context of intensified malaria control efforts. Enhanced supervision, increased testing, expanded interventions and an increased overall investment contributed to observed outcomes. However, the concurrent reduction in active malaria foci to zero by 2024 suggests that strengthened surveillance has played a key enabling role in achieving elimination milestones.

A central finding is that effectiveness depended on embedding the mobile app within an existing community-based health system. The VMW network, already responsible for frontline service delivery, provided a strong operational foundation, while the app enhanced their capacity through real-time reporting, structured workflows and feedback. This integration aligns local activities with national strategy and improves accountability, reinforcing the role of VMWs as both service providers and surveillance agents. High levels of user satisfaction, widespread adoption and sustained reporting performance further indicate that the system is acceptable and feasible at scale.

The choice of a dedicated mobile app, rather than a web-based platform, was critical in this context. Offline functionality, compatibility with low-cost devices and the ability to integrate features such as GPS and automated alerts addressed key operational constraints in remote areas. However, the findings also highlight that technology alone is insufficient. Continued training, supervision and system support were essential to maintain data quality and user confidence. Persistent knowledge gaps, particularly around data fields and reporting requirements, indicate that ongoing, targeted training remains necessary even in mature systems.

Implementation challenges were consistent with those reported in other low-resource settings, particularly connectivity limitations, intermittent electricity supply and variability in digital literacy. While offline data capture mitigated data loss, delays in synchronization remain a constraint. These findings underscore that digital solutions must be designed to operate under imperfect conditions and supported by responsive maintenance systems, such as device monitoring and replacement mechanisms.

Importantly, this study contributes to a limited evidence base on large-scale, nationally integrated mHealth systems. Unlike many pilot interventions, the Cambodian system is fully embedded within the national MIS, operates at scale and is sustained through domestic technical capacity. This local ownership appears to be a critical determinant of sustainability, with a reduced reliance on external partners enabling iterative system improvements and rapid troubleshooting. The strong performance observed here suggests that mHealth tools can move beyond pilot phases when they are contextually adapted, operationally integrated and institutionally supported.

This study has several limitations. First, findings were derived from routine surveillance data collected through the national MIS as part of programmatic implementation rather than a research study. As with all routine surveillance systems, reporting completeness and accuracy depend on the setting and operational capacity. Malaria is a mandatory notifiable disease in Cambodia’s public health system, and private-sector treatment has been banned since 2018. Thus, all cases are treated in the public health system and all treated cases are reported. However, cases failing to seek treatment would not be captured. Discovering these missing cases is being addressed through community engagement, targeted risk assessment and active case detection in remote populations [13,52–55]. In terms of ensuring accuracy, the VMW mobile app incorporates validation checks and supervisory review processes, and auditing shows the system complies with WHO guidance [38,39,50]. However, some degree of under-reporting, delayed reporting or data entry error may have occurred owing to the aforementioned operational constraints [39]. Second, the contextual and technical evaluation surveys were conducted as operational assessments and may be subject to selection bias as participation was self-determined. Thus, although participants had to be active VMWs and MMWs engaged with the surveillance system, the sample may not fully represent the experiences of all users nationally. However, survey responses were anonymized to minimize social desirability bias. Finally, the observational nature of this implementation study limits causal inference; improvements in surveillance performance occurred within the context of broader malaria elimination efforts, including strengthened supervision, training, program investments and intensification of malaria control interventions. Nevertheless, effective implementation of these initiatives required strengthened surveillance, supporting risk stratification and the timeliness and quality of case management as well as enabling efficient monitoring and evaluation of impacts.

In the malaria control phase, surveillance is employed for monitoring and evaluation to assess intervention impacts and large-scale risk stratification. In this case, aggregated passive detection through health facilities is sufficient to meet operational aims. However, when a country transitions to malaria elimination, surveillance becomes a core activity because every single asymptomatic or symptomatic infection serves as a reservoir for onward transmission. Finding and recording these cases often requires community-based surveillance.

Across the GMS, the transition from malaria control to elimination has driven significant investments in digital surveillance platforms. Myanmar launched the Malaria Case-Based Reporting app in 2018, later merging into a broader system alongside paper reporting, with a separate web-based platform for healthcare staff [20,45,46]. Thailand moved to web-based reporting in 2012 [56,57], adding an mHealth app in 2020 [58]. In Lao PDR, VMWs report via mobile phones to a dedicated web platform [59]. In 2021, Viet Nam introduced a web-based platform [60]. Common challenges include limited internet connectivity, electricity supply and technical support [20,57,59,60]. Increased access to smartphones has mitigated these issues somewhat. In contrast to Cambodia, in other GMS countries the private sector remains an important component of the care pathway and represents an additional challenge to achieving comprehensive surveillance [61].

Beyond the GMS, programs refocusing on malaria elimination are also considering mHealth interventions. In India, a pilot study of the FeverTracker mobile app demonstrated faster reporting and accessibility [49]; the app can also accept self-reports from the public and private sector [49]. Also in India, the Solution for Community Health-workers (SOCH) app uses self-validation features to improve data accuracy [47,48]. In Brazil, the centralized web-based SIVEP-Malaria system has an average reporting delay of 18 days, and mHealth solutions have been proposed to improve responsiveness to support elimination activities [62]. In Africa, many countries remain in the malaria control phase. In this context, mHealth tools are mainly used for periodic reporting from health facilities rather than community-level surveillance. One exception is Zambia, which piloted an SMS-based community surveillance system to support its 1-3-7 strategy. Though the intervention was well-received and improved case investigation rates, limited mobile coverage constrained its effectiveness [63].

For Cambodia, new challenges emerge as the country approaches malaria elimination and prepares for the post-elimination maintenance phase. VMWs play a central role in ‘last mile’ elimination activities, including screening for asymptomatic cases, door-to-door fever screening, targeted drug administration for individuals planning to enter forested areas and intermittent preventive treatment for high-risk forest goers [64]. In addition, mass drug administration and test-and-treat strategies are being piloted in selected regions. To maximize impact, these interventions must be context-specific, evidence-driven and well-coordinated. Effective surveillance continues to be pivotal in achieving these aims. The modular design of the MIS and associated apps allows the addition of data capture functions for pilot studies and ‘last mile’ interventions, supporting risk-based implementation, monitoring and evaluation. However, a key limitation is a lack of predictive or decision-support functionality. Integrating real-time data with machine learning and forecasting tools could significantly enhance resource allocation, planning and intervention effectiveness. Development is underway to embed such capabilities into the MIS, ensuring data-driven decision-making at all operational levels aligned with evolving priorities.

The post-elimination role of VMWs must also be considered. Achieving and maintaining WHO malaria-free certification requires a robust surveillance system capable of rapidly identifying, geolocating and investigating every malaria case to classify autochthonous versus imported cases and prevent re-establishment of transmission [38]. However, a key challenge is maintaining an engaged and functional surveillance network as malaria cases decline and local transmission ceases [65,66]. To support this transition, Cambodia is expanding VMW responsibilities beyond malaria. The VMW mobile app has been adapted to support combined diagnostics for malaria along with C-reactive protein (a biomarker for bacterial infection) or dengue [67]. This positions VMWs as the first point of contact for febrile illness, increasing the likelihood that malaria cases are detected early [67]. Communities have also expressed interest in expanding VMW roles to address common conditions such as headaches, gastrointestinal complaints and chronic disease monitoring (e.g., hypertension, diabetes) [66]. These services align with national health priorities and, if integrated into the app and linked to the national health information system, could enhance access to care in underserved areas. Nevertheless, expanding the VMW scope introduces challenges such as time constraints, appropriate incentives, community acceptability and long-term sustainability. Careful consideration of these factors will be necessary to preserve the effectiveness of VMW malaria surveillance while supporting broader health system goals in a post-elimination era.

## Conclusion

Progress toward malaria elimination in Cambodia is supported by the availability of timely, geolocated, case-based surveillance capable of detecting and responding to residual transmission foci. The VMW mobile application, designed for use within a community-based health system, facilitates near real-time data capture and reporting at the point of care. Its implementation has been associated with improvements in reporting timeliness and completeness, alongside strengthened visibility of cases at the national level, supporting more responsive programmatic operations.

More broadly, mobile-based reporting represents a promising approach to strengthening malaria surveillance, particularly in settings with established community health networks. Persistent challenges include connectivity constraints, variability in digital literacy and the need for sustained technical and supervisory support. Long-term utility depends on continued integration with national strategies and the capacity of local teams to manage, adapt and maintain these systems as epidemiological and operational conditions evolve.

In the GMS, where malaria transmission is increasingly focal and concentrated in hard-to-reach populations, surveillance assumes a central role in elimination efforts. As transmission declines, the ability to detect, classify and respond to every case becomes increasingly important for preventing onward transmission and resurgence. In this context, surveillance functions not only as a monitoring tool but as a core operational component of elimination strategies.

Sustaining these systems remains a key challenge, particularly in the context of declining global and regional funding. Cambodia’s MIS, developed using open-source architecture and maintained by a small, in-country technical team, illustrates a potentially sustainable approach, reducing reliance on external vendors while enabling iterative system development. This experience suggests that locally developed and managed digital surveillance systems may offer a viable pathway for strengthening community-based disease surveillance in resource-constrained settings.

## Supporting information

S1 File Screenshots of the Cambodian Village Malaria Worker application (VMW app), explaining functionality and content. (PDF)
