# Supplementary material for: Community-based malaria surveillance in Cambodia using a mobile reporting app for village malaria workers: An implementation case study
Source: PLOS Digit Health. 2026 Sep 3;5(9):e0001626. doi: 10.1371/journal.pdig.0001626 (PMC13541183; doi:10.1371/journal.pdig.0001626)
Supplement: Supplementary file 1 [file pdig.0001626.s001.pdf]

Supporting information

Community-based malaria surveillance in Cambodia using a mobile reporting app for village malaria workers: an implementation case study

Pengby Ngor, et al.

S1 File. Screenshots of the Cambodian Village Malaria Worker application (VMW app), explaining functionality and content.

This appendix includes screenshots of the Cambodian Village Malaria Worker application (VMW app), explaining functionality and content.

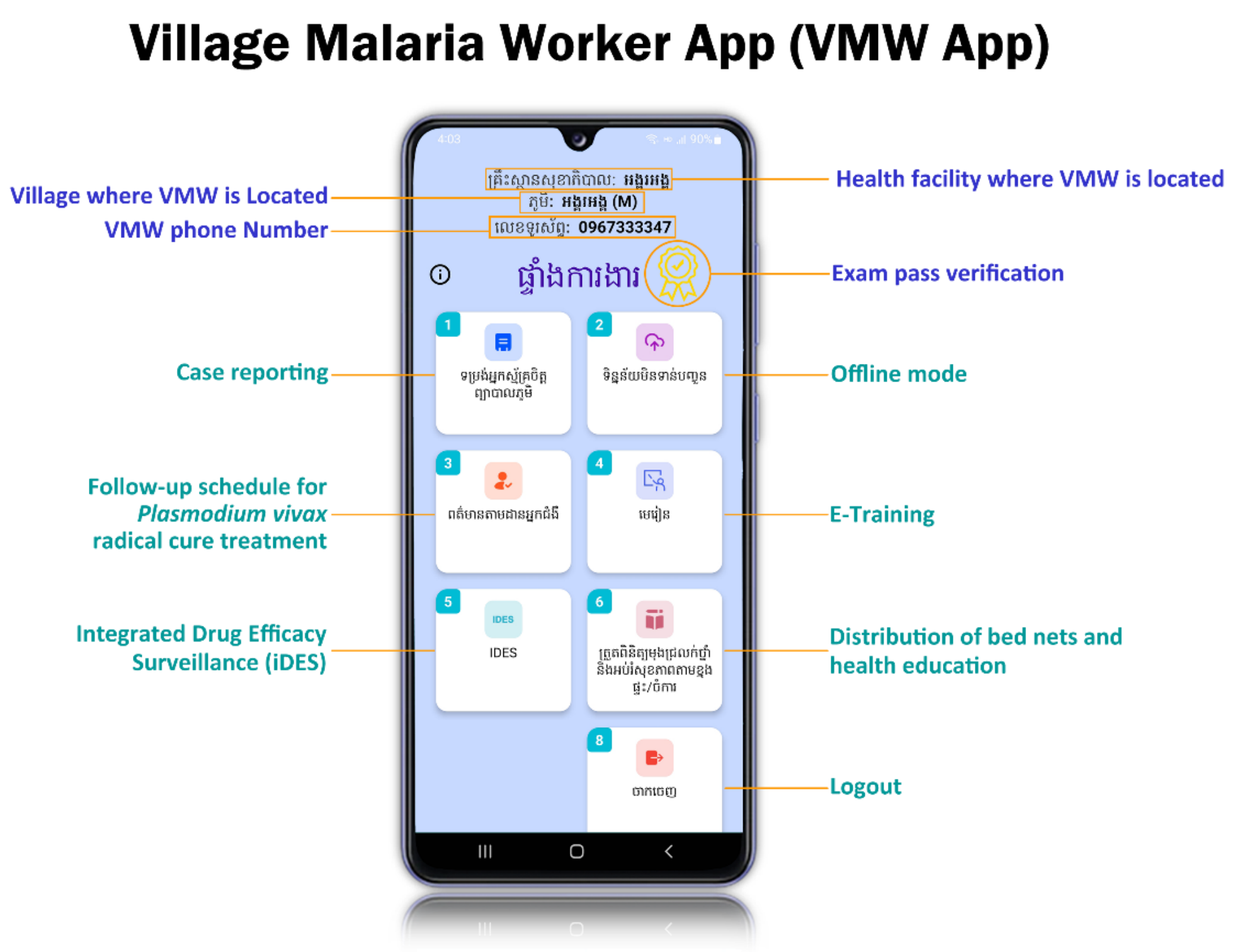

Case Reporting (VMW Form)

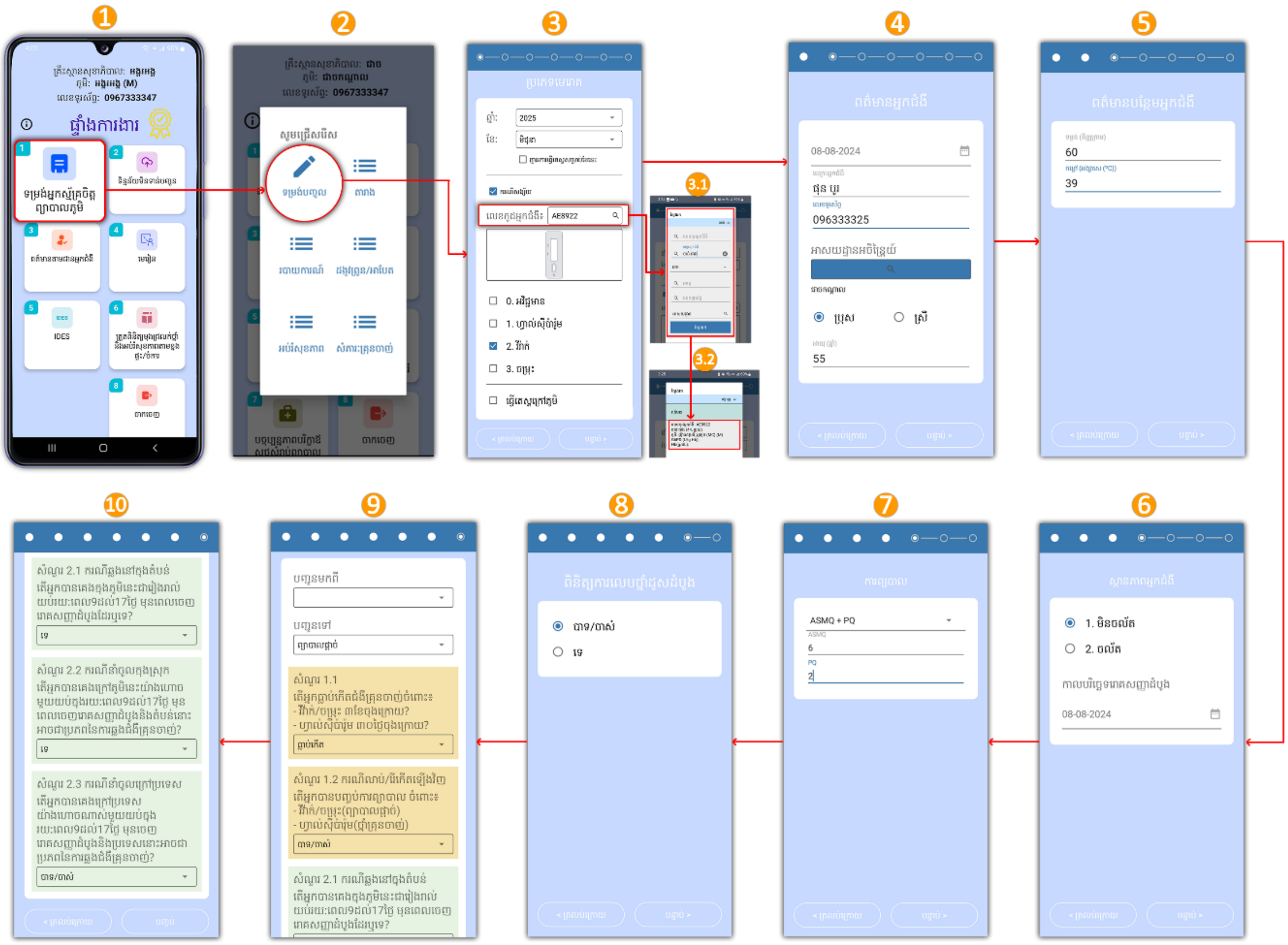

1. *Village Malaria Worker App home menu*
2. *Menu for data entry, such as case reporting, table, report, education, malaria commodity*
3. *Choose the month of the report for data entry, tick for suspected malaria, choose the result of test and take picture of RDT*

   3.1 *Search patient by patient code, name, sex, age, phone number and address*

   3.2 *See patient code, name, age, sex, address, G6PD and hemoglobin*
4. *Patient information such as date for blood test, name, address, sex and age*
5. *More detail about the patient, such as weight and temperature*
6. *Patient situation, such as non-mobile, mobile and date of first symptom*
7. *Treatment, such as name of ACT, number of ACT and number of primaquine*
8. *Check the first dose has been observed (yes, no)*
9. *Referral from (none, private sector, other) and referral to (P. vivax radical cure, severe, other).*
10. *Classification Q1.1 and 1.2 for relapse/recrudecent, Q2.1 for locally aquired, Q2.2 for domestically Imported, Q2.3 for internationally imported*

Patient Unique Identification

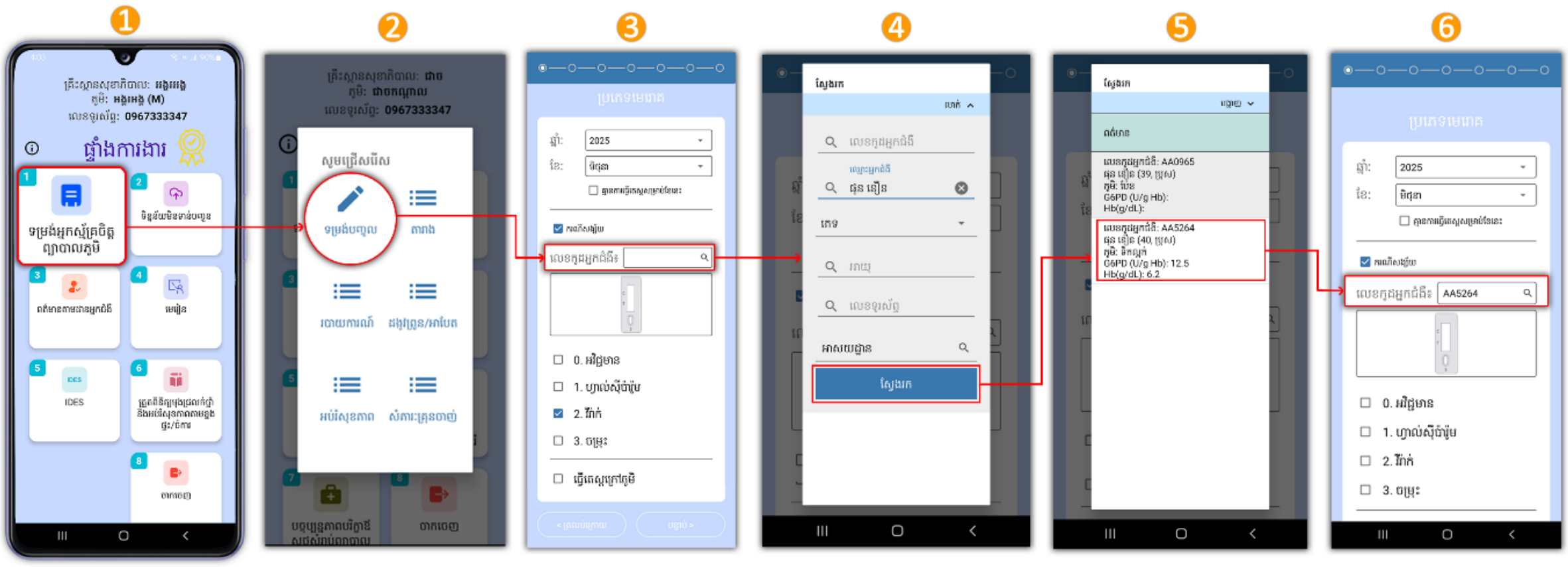

1. Village Malaria Worker app home menu
2. Menu for data entry, such as case reporting, table, report, education, malaria commodity
3. Choose the month of the report for data entry, tick for suspected case to choose patient code
4. Search for the patient by patient code, name, sex, age, phone number and address
5. See patient code, name, age, sex, address and result of G6PD and hemoglobin
6. Patient code is selected

E-Training

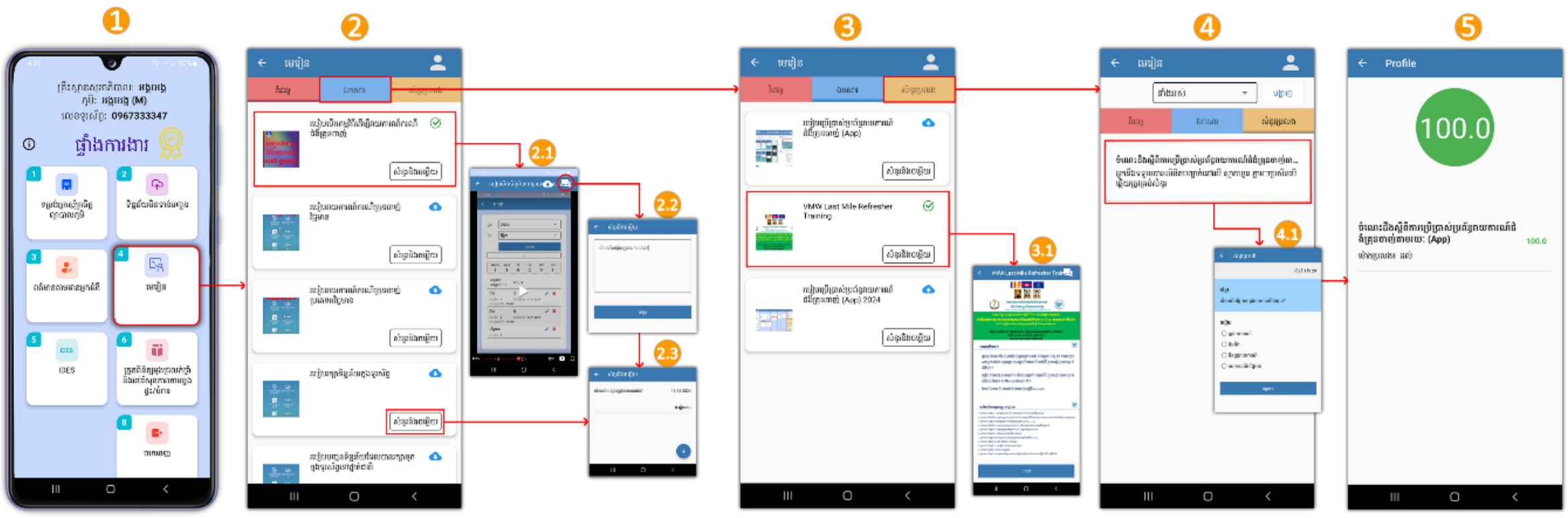

1 Village Malaria Worker App home menu
2 First menu for E-Training is for video and allows downloads of videos to store in the phone for offline viewing
2.1 Play video
2.2 Questions after the video has been watched plus the opportunity for questions and clarifications
2.3 Question and answer (MIS team replies to questions) and ability to create questions
3 Second menu for E-Training is for documentd which can be downloaded and stored in the phone for offline use
3.1 View document
4 Third menu for E-Training is the quiz for knowledge examination to obtain the exam pass verification
4.1 Questionnaire form
5 Exam results

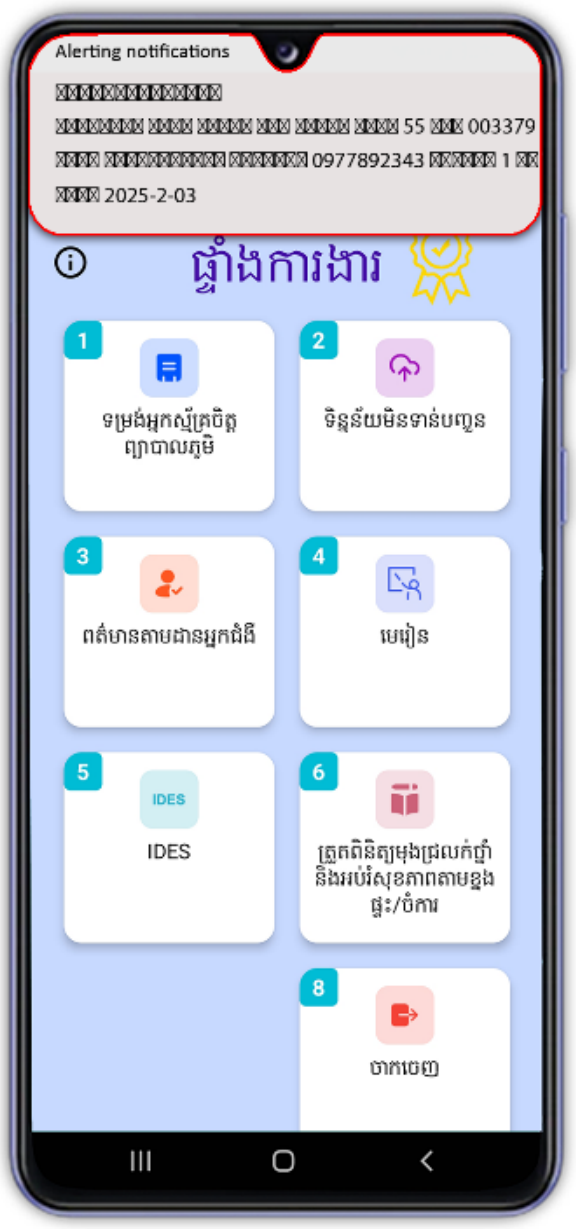

Reminder for *Plasmodium vivax* radical cure follow-up

- *Reminder notification for Plasmodium vivax/mix radical cure follow-up, including patient details (name, sex, age, unique ID, village, and phone number).*
- *The scheduled follow-up for Day 1 is on 03 February 2025.*

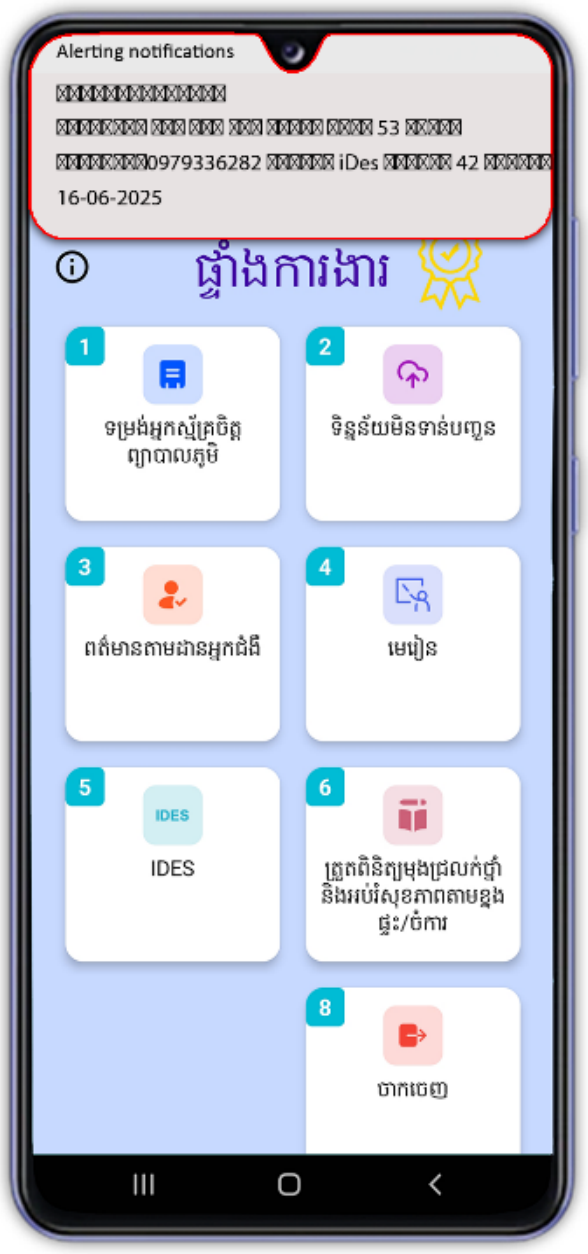

Reminder for Integrated Drug Efficacy Surveillance Follow-up

- *Reminder notification for iDES follow-up, including patient details (name, sex, age, unique ID, village, and phone number).*
- *The scheduled follow-up for Day 42 is on 16 June 2025.*

Follow-up Schedule for *Plasmodium vivax* Radical cure

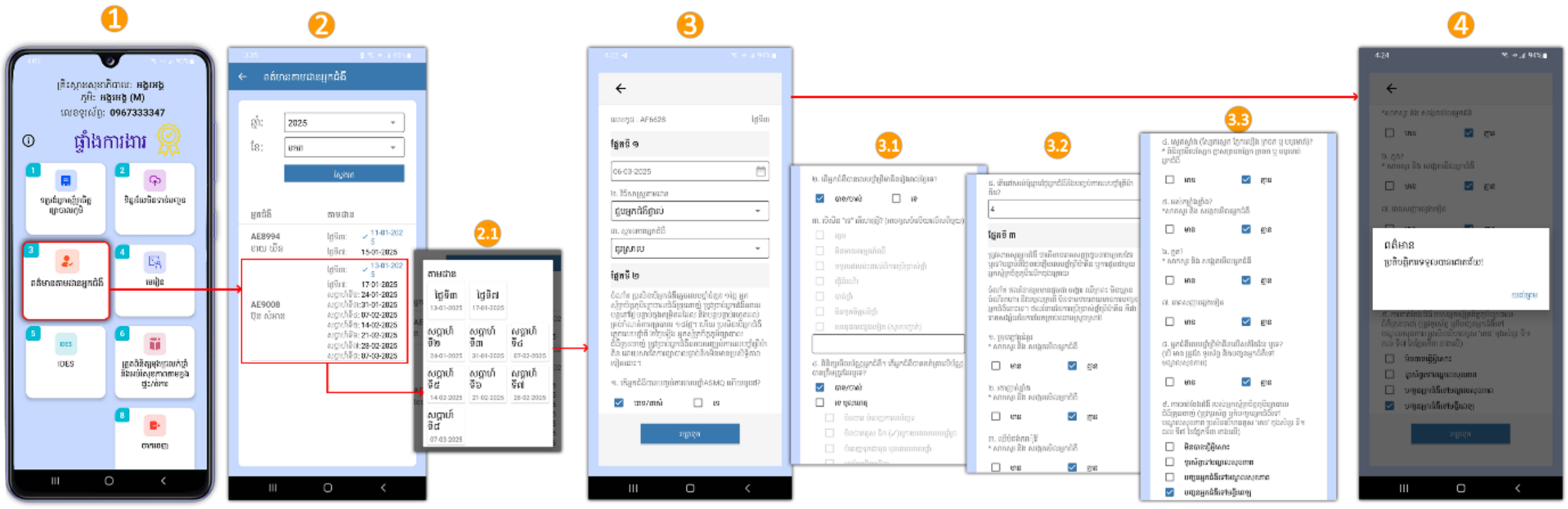

1. *Village Malaria Worker App home menu*
2. *Select year and month, touch 'Search' to show patient information and schedule for follow-up*
2.1 *Follow-up days and date to enter follow-up data*
3. *Questionnaire part 1: method for follow-up (in person or phone call) and patient status (recovered, unchanged, more severe)*
3.1 *Part 2: record administration of artesunate-amodiaquine and primaquine and if patient noted taking the medicine on the patient card*
3.2 *Part 3: malaria symptoms following medication administration: severe fever, severe shivering, sore throat with irritation...*
3.3 *pale and weak, severe fatigue, vomiting, other symptoms and overdose; for each symptom present actions are given (nothing or refer)*
